# Comparative gene expression analysis of *Porphyromonas gingivalis* ATCC 33277 in planktonic and biofilms states

**DOI:** 10.1371/journal.pone.0174669

**Published:** 2017-04-03

**Authors:** P. Romero-Lastra, MC. Sánchez, H. Ribeiro-Vidal, A. Llama-Palacios, E. Figuero, D. Herrera, M. Sanz

**Affiliations:** 1 Laboratory of Dental Research, University Complutense, Madrid, Spain; 2 ETEP (Etiology and Therapy of Periodontal Diseases) Research Group, University Complutense, Madrid, Spain; East Carolina University Brody School of Medicine, UNITED STATES

## Abstract

**Background and objective:**

*Porphyromonas gingivalis* is a keystone pathogen in the onset and progression of periodontitis. Its pathogenicity has been related to its presence and survival within the subgingival biofilm. The aim of the present study was to compare the genome-wide transcription activities of *P*. *gingivalis* in biofilm and in planktonic growth, using microarray technology.

**Material and methods:**

*P*. *gingivalis* ATCC 33277 was incubated in multi-well culture plates at 37°C for 96 hours under anaerobic conditions using an *in vitro* static model to develop both the planktonic and biofilm states (the latter over sterile ceramic calcium hydroxyapatite discs). The biofilm development was monitored by Confocal Laser Scanning Microscopy (CLSM) and Scanning Electron Microscopy (SEM). After incubation, the bacterial cells were harvested and total RNA was extracted and purified. Three biological replicates for each cell state were independently hybridized for transcriptomic comparisons. A linear model was used for determining differentially expressed genes and reverse transcription quantitative polymerase chain reaction (RT-qPCR) was used to confirm differential expression. The filtering criteria of **≥** ±2 change in gene expression and significance p-values of <0.05 were selected.

**Results:**

A total of 92 out of 1,909 genes (4.8%) were differentially expressed by *P*. *gingivalis* growing in biofilm compared to planktonic. The 54 up-regulated genes in biofilm growth were mainly related to cell envelope, transport, and binding or outer membranes proteins. Thirty-eight showed decreased expression, mainly genes related to transposases or oxidative stress.

**Conclusion:**

The adaptive response of *P*. *gingivalis* in biofilm growth demonstrated a differential gene expression.

## Introduction

Human dental plaque is a complex and dynamic biofilm attached to tooth surfaces, where microbial communities are embedded in a matrix of bacterial extracellular polymeric substances (EPS), proteins, salivary peptides and food scraps [1, 2]. The differential activity of these microbial communities within the dental biofilm may have profound implications in the onset and progression of periodontitis, one of the most prevalent chronic inflammatory diseases affecting humans [3]. *Porphyromonas gingivalis*, a Gram-negative and black-pigmented anaerobic bacterium is one of the keystone pathogens associated with the etiology of periodontitis. Its main ecological niche is the oral microbiome [4] and its pathogenic activity has been directly related to its relative high numbers and proportions within the subgingival biofilm, as well as the expression of virulence factors that facilitate its colonization within the periodontal tissues and its resistance from the host inflammatory and immune responses. [5–7].

Virulence factors in periodontal pathogens have been attributed to either presence of highly pathogenic strains or to the up- and down- regulation of a number of genes due to the specific ecological conditions of the bacterial communities within the biofilm. In fact, several transcriptomic studies have been conducted to elucidate the behavior of different pathogenic bacteria growing in biofilm [8–11]. Whiteley *et al*. [10] reported that about 1% of the genes from *Pseudomonas aeruginosa* had shown differential expression when growing in biofilm compared with planktonic. Liu *et al*. [9] reported that 16.2% of the genes from *Clostridium acetobutylicum* were differentially expressed in biofilm growth, mainly up-regulation of genes involved in amino acid biosynthesis, sporulation, extracellular polymer degradation and other various metabolic processes, what indicated that *C*. *acetobutylicum* had a distinct phenotype when growing in a biofilm.

Similarly, transcriptomic studies have reported that approximately 18.0% of the W50 genome of *P*. *gingivalis* was differentially expressed in biofilms [8]. These studies have shown down-regulation of genes encoding for cell envelope biogenesis, DNA replication, energy production and biosynthesis of co-factors and up- regulation of genes involved in transport and binding proteins. Some of these studies have focused specifically on LuxS-dependent signaling and quorum-sensing-regulated genes since they play an important role in the physiology of these micro-organisms, their communication with other bacteria, and their adaptation to the biofilm environment [12–14]. Yamamoto *et al*. [15] reported that an increase of more than 1.5-fold in the number *P*. *gingivalis* (ATCC 33277) genes differentially regulated during the biofilm growth (312/2,090 genes, 155 genes were up-regulated and 157 genes were down-regulated).

In spite of these studies, our understanding of the regulatory processes and interactions, which allow *P*. *gingivalis* to grow within the biofilm and to develop its virulence is still limited. It is, therefore, the aim of this study to assess the differential expression of *P*. *gingivalis* genes under two different physiological states, planktonic and biofilm growth, using transcriptomic analysis in an *in vitro* static model.

## Material and methods

### Bacterial strain

Standard reference strain *P*. *gingivalis* ATCC 33277 was selected for the present study. Bacteria were grown on blood agar plates (Blood Agar Oxoid No 2; Oxoid, Basingstoke, UK), supplemented with 5% (v/v) sterile horse blood (Oxoid), 5.0 mg/L hemin (Sigma, St. Louis, MO, USA) and 1.0 mg/L menadione (Merck, Darmstadt, Germany) in anaerobic conditions (10% H_2_, 10% CO_2_, and balance N_2_) at 37°C for 72 hours.

### Bacterial growth and experimental assays

Planktonic cultures of *P*. *gingivalis* were grown anaerobically at 37°C for 24 h in a protein-rich medium containing brain-heart infusion (BHI) (Becton, Dickinson and Company, Franklin Lakes, NJ, USA) supplemented with 2.5 g/L mucin (Oxoid), 1.0 g/L yeast extract (Oxoid), 0.1 g/L cysteine (Sigma), 2.0 g/L sodium bicarbonate (Merck), 5.0 mg/L hemin (Sigma), 1.0 mg/L menadione (Merck) and 0.25% (v/v) glutamic acid (Sigma). Upon reaching late-exponential phase [10^9^ colony forming units (CFU)/mL, as measured spectrophotometrically by optical density at 550 nm], the cells were diluted in modified BHI medium to obtain a final concentration of 10^8^ CFU/mL.

In order to study the gene expression of *P*. *gingivalis*, in biofilm or planktonic growth, under the same culture conditions, a volume of 1.5 mL of *P*. *gingivalis* inoculums was placed in pre-sterilized polystyrene 24-well tissue culture plates (Greiner Bio-one, Frickenhausen, Germany) with or without the presence of sterile ceramic calcium hydroxyapatite discs (HA) [7-mm diameter (standard deviation, SD = 0.2) and 1.8 mm thickness] (Clarkson Chromatography Products, Williamsport, PA, USA). To carry out the experiment, a total of 45 multiwell plates were used. In each plate 19 wells were filled with disk to develop the biofilms (each of the aggregates in each hydroxyapatite disk is considered as a biofilm) and the other five wells were used to analyze the planktonic state without hydroxyapatite disk.

Plates were incubated in anaerobic conditions at 37°C for 96 h. Wells containing only culture medium were also incubated to verify sterility and the possible contamination of bacteria growing in both planktonic and biofilm growth was frequently checked.

### Confocal Laser Scanning Microscopy (CLSM) analysis to monitor *P*. *gingivalis* biofilm development

To ensure the change of *P*. *gingivalis* phenotype, from planktonic to biofilm, its growth was studied by CLSM when the biofilm reached a mature state (from 24 to 96 h). To confirm the reproducibility of the biofilm-growth, three independent experiments using trios of biofilms were carried out for each incubation time (a concentration of 10^8^ CFU/mL *P*. *gingivalis* cells in planktonic culture were placed on sterile hydroxyapatite discs). Before the CLSM analysis, the discs were rinsed in 2 mL of sterile Buffer Phosphate Saline (PBS) three times (10 sec of immersion time per rinse), in order to remove non-adherent bacteria. Non-invasive confocal imaging of fully hydrated biofilms was carried out using a fixed-stage Ix83 Olympus inverted microscope coupled to an Olympus FV1200 confocal system and with a ×63 water-immersion lens (Olympus; Shinjuku, Tokio, Japan). Specimens were stained with LIVE/DEAD^®^ BacLightTM Bacterial Viability Kit solution (Molecular Probes B. V., Leiden, The Netherlands) at room temperature. A 1:1 fluorocromes ratio and 9±1 min of staining time was used to obtain the optimum fluorescence signal at the corresponding wave lengths (Syto9: 515–530 nm; PI: >600 nm). At least three separate and representative locations on the HA discs covered with biofilm were selected for the study. The CLSM control software was set to take a z-series of scans (xyz) of 0.5 μm thickness (8 bits, 1024x1024 pixels). Image stacks were analyzed with the proprietary Olympus^®^ software (Olympus).

### Scanning Electron Microscopy (SEM) analysis

Before SEM analysis, three hydroxyapatite discs covered with biofilms grown *in vitro* for 96 h were fixed in 4% paraformaldehyde and 2.5% glutaraldehyde for 4h at 4°C. Then, the discs were washed twice in PBS and sterile water (immersion time 10 min) and then, dehydrated through a series of graded ethanol solutions (50, 60, 70, 80, 90 and 100%; immersion time per series, 10 min), Then, the samples were critical point dried, sputter-coated with gold and analysed with an scanning electron microscope JSM 6400 (JSM6400; JEOL, Tokyo, Japan) equipped with back-scattered electron detector and with an image resolution of 25 KV.

### Harvesting of planktonic and biofilm cells for gene expression analysis

After 96 h of incubation, *P*. *gingivalis* planktonic and biofilm cells were harvested (three biological replicates of each state) for independent hybridization.

For planktonic cells 1 mL was recovered from 15 diskless well. In the same experiments a set of 300 biofilms were harvested independently, then added to 1 mL of sterile PBS, disaggregated by vortexing during 3 min. In both cases the samples were recovered as partial plucks by centrifugation at 9,000 rpm at 4°C during 5 min, in order to obtain a final 10 μg of total RNA for each replicate in each state. To preserve the bacterial total RNA intact during the time taken for the procedures, the work has always been in cold conditions.

In all cases, after the incubation period, an aliquot of each sample and 1 to 3 discs were used as quality control. They were cultivated on supplemented blood agar plates under anaerobic conditions at 37°C during two weeks to assure the absence of contamination.

### Total RNA extraction

Total RNA was extracted from the harvested samples using the TRIzol^®^ Max Bacterial RNA Isolation Kit (Ambion, Life Technologies, Carlsbad, CA, USA). Briefly, pools from planktonic and biofilm growth samples were suspended in 200 μL of preheated Max bacterial Reagent^®^ (Ambion), incubated at 95°C for 4 min and then chilled on ice for 10 min. After, 1 mL of TRIzol^®^ reagent (Ambion) was added to lysate the cells, incubating them at room temperature 5 min. After that, 200 μL of cold chloroform was added and incubated at room temperature for 3 min. The mixtures were then centrifuged at 13,000 rpm for 15 min at 4°C. RNA colourless aqueous phase (~ 600 μL) was collected, augmented with 0.5 mL of cold isopropanol, mixed by inversion, and incubated at room temperature for 10 min. After centrifugation at 13,000 rpm for 10 min at 4°C, the pellet of RNA was suspended in 1 mL of cold 75% ethanol, centrifuged at 9,000 rpm for 5 min, air-dried and suspended in 50 μL of RNase-free water (Roche Diagnostics, Mannheim, Germany). The samples were then treated with DNase I (Ambion, NY, USA) to remove any contaminating DNA (set of RNase-free DNase; Qiagen, CA, USA) and purified using columns of RNeasy Mini kit (Qiagen) according to the manufacturer's protocol.

RNA quantity was measured by NanoDrop ND1000 spectrophotometer (NanoDropTechnologies; Thermo Scientific^™^, LLC, Wilmington, DE, USA). RNA quality was monitored by Agilent 2100 Bioanalyzer (Agilent Technologies, Santa Clara, CA, USA). All the samples used in this study exhibited an A260/A280 ratio of at least 2.0.

### cDNA synthesis and transcriptomic analysis

Three biological replicates were independently hybridized for each transcriptomic comparison. Fluorescently labeled cDNA for microarray hybridizations was obtained by using the SuperScript Indirect cDNA Labeling System (Invitrogen). In brief, 5 μg of total RNA was transformed to cDNA with Superscript III reverse transcriptase using random hexamers as primers and with aminoallyl-modified nucleotides in the reaction mixture. After cDNA purification, the Cy3 fluorescent dyes (Amersham Biosciences) were coupled to the amino-modified first-strand cDNA. Labelling efficiency was assessed using a NanoDrop ND1000 spectrophotometer (NanoDropTechnologies).

Preparation of probes and hybridization was performed as described (One-Color Microarray Based Gene Expression Analysis Manual Ver. 6.5, Agilent Technologies). Briefly, for each hybridization, 600 ng of Cy3 probes were mixed and added to 5 μL of 10x Blocking Agent and Nuclease free water in a 25 μL reaction. Then, 25 μL from 2x GExHybridization buffer was added and mixed carefully. The samples were placed on ice and quickly loaded onto arrays, hybridized at 65°C for 17 h and then washed once in GE wash buffer 1 at room temperature (1 min) and once in GE Wash Buffer 2 at 37°C (1 min).

Slides corresponded to Agilent *P*. *gingivalis* Oligo Microarrays 8x15K (074976), a genome annotation specific for strain ATCC 33277 and W83. For each culture pair, three technical replicates of array hybridizations were performed.

### Microarray and data analysis

Images from Cy3 channel were equilibrated and captured with a high-resolution scanner (Agilent) and spots quantified using Feature Extraction software (Agilent). Background correction and normalization of data expression were performed using LIMMA [16, 17]. LIMMA is part of bioconductor, an R language project [18]. For local background correction and normalization, the methods "normexp" and loess in LIMMA were used, respectively [16]. To ensure similar distribution across arrays and to achieve consistency among arrays, log-ratio values were scaled using the median-absolute-value as scale estimator [17].

Linear model methods were used for determining differentially expressed genes. Each probe was tested for changes in expression over replicates by using an empirical Bayes moderated t-statistic [17]. To control the false discovery rate *p-values* were corrected by using the method of Benjamani and Hochberg [16, 17]. The expected false discovery rate was controlled to be less than 5% and a filtering criterium of increase/decrease up to 2-fold differential expression between states was selected.

The National Center for Biotechnology (Genomics Unit) at Universidad Autónoma, Madrid (Spain) performed the hybridizations and statistical analysis.

### Assessment of microarray data by Reverse Transcription-quantitative Polymerase Chain Reaction (RT-qPCR)

To confirm the microarray results using RT-qPCR, nine genes differentially expressed between both situations were selected, four genes from the up-regulated group and five from the down-regulated one. Specific primers were designed using the Universal Probe Library Roche software tool (Roche Diagnostics) (Table 1). All quantifications were normalized to the *P*. *gingivalis* 16S rRNA gene.

**Table 1:** Primers used for Reverse Transcription-quantitative Polymerase Chain Reaction (RT-qPCR).

LOCUS NAME	PUTATIVE IDENTIFICATION	PRIMER SEQUENCES
**porP**	Porins	Forward 5´-3´: GGGTAGTGACCGAAACGAGA
		Backward 5´-3´: GAAGGCATATTGCCCCATC
**PGN 0319**	Probable RNA polymerase sigma-70 factor ECF subfamily	Forward 5´-3´: CGTCTGGTGGAAGCTGCTAT
		Backward 5´-3´: CAGCCGGAAAGTCATTCG
**PG 0215**	Hypothetical protein	Forward 5´-3´: GCCTTCGATGCTGTATCCAT
		Backward 5´-3´: TCAAAGGTCGAAAAGCTCCT
**PGN 0320**	Hypothetical protein	Forward 5´-3´: GCCTTCGATGCTGTATCCAT
		Backward 5´-3´: TCAAAAGGTCGAAAAGCTCCT
**PG 2130**	Hypothetical protein	Forward 5´-3´: TTCGAATGTGCCAAGTGC
		Backward 5´-3´: TCGTCACACCGAAGTAGTCG
**PGN 0575**	Transposase in ISPg1	Forward 5´-3´: AGACAATCGGAGCGAGGAG
		Backward 5´-3´: TTTACGCYGACGGACAACCT
**PGN 1925-Cas1**	Mobile and extrachromosomal element functions	Forward 5´-3´: GAGCCTCTCTCCAACGCTATC
		Backward 5´-3´: GCCCTCCGCTATGGGTAT
**PG 0619**	Alkyl hydroperoxide reductase, F subunit	Forward 5´-3´: CTGCAGCCATCYATTCTGCTC
		Backward 5´-3´: CTACCCGTTCGGCTACGAT
**vimF**	Virulence modulating gene F	Forward 5´-3´: CCGAAATTCTCCGCCATAG
		Backward 5´-3´: CTCCGGGCTTCTCTGTGTT

To carry out the Reverse Transcription-qPCR, cDNA was generated from 1 μg of total RNA using the High Capacity cDNA Archive Kit (Applied Biosystems, ThermoFisher Scientific) in a 10 μL of final reaction volume. After that, quantitative PCR reactions were performed in triplicate by using 5 μL per well of each cDNA, and 3 μL of a mix composed by 0.4 μM of each primer, 5x HOT FIREPol^®^ EvaGreen^®^ qPCR Mix Plus (ROX), and nuclease-free water, to reach a final volume of 8 μL in 384-well optical plates. PCR reactions were run in an Applied Biosystems ABI PRISM 7900HT machine with SDS v2.4 software and standard protocol from Applied Biosystems (95°C 10 min, 40 cycles of 95°C 15 sec and 60°C 60 sec, and a final standard dissociation protocol). The results were analysed with the Comparative Ct Method (ΔΔCt) [19].

## Results

**Fig 1:**
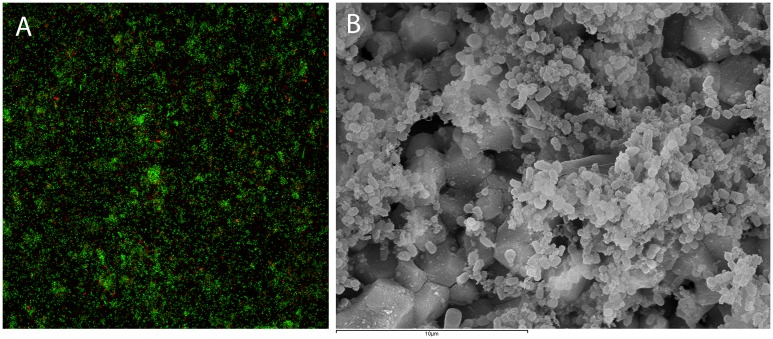
Representative confocal (A) and scanning electron (B) micrographs representing *Porphyromonas gingivalis* ATCC 33277 biofilm after 96h of growth. BacLight Live/Dead strain was used to assess the viability of cells in CLSM distinguishing viable bacteria depicted as green and non-viable as red stained cells.

CLSM and SEM confirmed that *P*. *gingivalis* ATCC 33277 changed its phenotypic state, from planktonic to a mono-species biofilm. Fig 1 shows representative CLSM (depicting viable bacteria as green and nonviable as red stained cells.) and SEM images of the obtained biofilms at 96 h of incubation,

With the use of the filtering criteria threshold of two-fold change in differential expression (up or down) of the contained in *P*. *gingivalis* ATCC 33277 arrays, a total of 92 out of 1,909 (4.8%) genes were differentially expressed in the biofilm phenotype compared to planktonic growth. These differences were statistically significant (p<0.05).

**Fig 2:**
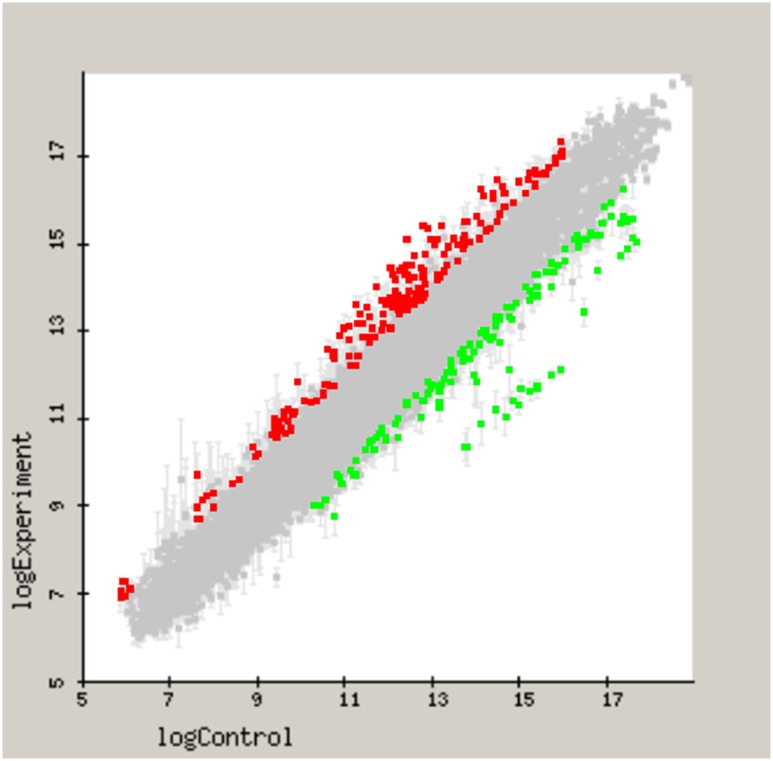
Differential gene expression in *Porphyromonas gingivalis* ATCC 33277 biofilm as opposed to planktonic cells. Differentially expressed genes with 2.0 fold change (up or down) and p-value < 0.05 were plotted. X-axis presents fold difference between log expression of planktonic, and y-axis shows the log expression of biofilm. Up-regulated genes (over-expressed in biofilm) were represented as red color and down-regulated genes were colored in green.

Fig 2 shows the genes differentially expressed in *P*. *gingivalis* ATCC 33277 biofilms compared to planktonic cells. From the identified genes, the 54 up-regulated genes in the biofilm were mainly related to cell envelope, transport and binding proteins, outer membranes proteins, DNA repair enzymes, ribosomal proteins, or genes related to transcription initiation. Conversely, the 38 genes that were down-regulated in biofilm cells were mostly genes encoding proteins related to transposases, the CRISPRs system (cluster regularly inter-spaced short palindromic repeats) or oxidative stress.

In Table 2, these genes are grouped by functional categories, such as the genes encoding for the cationic outer membrane proteins (OmpH-1 and OmpH-2, PG_0448, and PG_0987), which have shown up-regulated expression in this model of *P*. *gingivalis* biofilm. These genes codifying proteins located specifically in the outer membrane vesicles, have been recognized as important virulence factors of *P*. *gingivalis*. Moreover, the gene coding lipoprotein PGN_0151 appeared up regulated by a factor of 3.16 (SD 0.28) compared to planktonic state (Table 2). Similarly, the genes related with the Por Secretion System (PorSS) (*porP*, PGN_1514 and PG_0448), involved in the biosynthesis of cell surface polysaccharides and implicated in the translocation of gingipains were up-regulated in biofilm growth. These proteins are well known virulence factors and serve as anchors for Rgp, Kgp, hemagglutinins, and the hemoglobin receptor protein. Only one gene, implicated in predicted exporter proteins (PGN_0946) was found significantly down regulated.

**Table 2:** Genes differently expressed in *Porphyromonas gingivalis* ATCC 33277 biofilm (cutoff ratio ≥ ±2.0 fold change, p-value < 0.05) for the microarray analysis, grouped by functional role categories.

LOCUS NAME	PUTATIVE IDENTIFICATION^A^	AVG RELATIVE FOLD CHANGE (SD)^B^
**1. GENES RELATED TO CELL ENVELOPE**		
**ompH-1**	Cationic outer membrane protein OmpH	2.38 (0.37)
**ompH-2**	Cationic outer membrane protein OmpH	2.23 (0.09)
**PG 0987**		2.85 (0.12)
**PGN 0301**		2.17 (0.08)
**PGN 0968**		3.29 (0.12)
**PGN 0151**	Lipoprotein	3.16 (0.28)
**PGN 0946**	Predicted exporter protein	-2.29 (0.23)
***porP***	Porins	2.43 (0.27)
**PGN 1514**	Conserved hypothetical proteinporins	2.09 (0.09)
**PG 0448**	Porins	2.54 (0.35)
**2. GENES RELATED TO OXIDATIVE STRESS AND METABOLISM**		
**PGN 2076**	Bacterioferritin-associated ferredoxin proteins	-2.46 (0.33)
**PG 2213**	Bacterioferritin-associated ferredoxin proteins	-3.50 (0.39)
**PG 2029**	Metalloprotease	2.22 (0.16)
**PGN 1120**	Putative NADPH-NAD transhydrogenase	2.26 (0.05)
**PGN 1122**	NADPH-NAD transhydrogenase beta subunit	2.43 (0.30)
***pntB***	NAD(P) transhydrogenase, beta subunit	2.35 (0.28)
**PG 0618**	Alkyl hydroperoxide reductase, C subunit	-5.52 (1.98)
**PGN 0660**	Putative alkyl hydroperoxide reductase C subunit	-4.93 (1.21)
**PG 0619**	Alkyl hydroperoxide reductase, F subunit	-13.13 (0.70)
**PGN 0661**	Alkyl hydroperoxide reductase F subunit	-11.59 (1.16)
**3. GENES RELATED TO TRANSPOSON FUNCTIONS**		
**PGN 0219**	Partial transposase in ISPg1	-2.78 (0.29)
**PGN 0575**	Transposase in ISPg1	-2.50 (0.35)
**PGN 1216**	Transposase in ISPg1	-2.43 (0.26)
**PGN 1420**	Transposase in ISPg1	-2.47 (0.16)
**PGN 0478**	Partial transposase in ISPg4	2.16 (0.14)
**PGN 0578**	Conserved hypothetical protein found in conjugate transposon	2.12 (0.14)
**PGN 0579**	Conserved hypothetical protein found in conjugate transposonTra related domains	-2.83 (0.55)
**4. GENES RELATED TO CRISPR**		
**PGN 1924-Cas2**	Mobile and extrachromosomal element functions	-2.10 (0.06)
**PGN 1925-Cas1**	Mobile and extrachromosomal element functions	-2.53 (0.42)
**5. GENES RELATED TO LYSOZYMES**		
**PGN 1286**	Probable lysozyme	2.63(0.28)
**6. GENES RELATED TO FIMBRIA**		
***fimD***	Minor component FimD	-2.30 (0.26)
**7. GENES RELATED TO RIBOSOME**		
***rpmHrpmH***	50S ribosomal protein L34 ATCCRibosomal protein L34 W83	2.41 (0.26) 2.47 (0.34)
***rpsF***	30S ribosomal protein S6	2.32 (0.22)
***rpIl***	50S ribosomal protein L9	2.18 (0.09)
***KsgA***	Dimethyladenosine transferase	-2.24 (0.06)
**8. GENES RELATED TO TRANSCRIPTION INITIATION RNA POLYMERASE SIGMA-70 FACTOR, ECF SUBFAMILY**		
**PG 0214**	RNA polymerase sigma-70 factor, ECF subfamily	4.37 (0.18)
**PG 0985**	RNA polymerase sigma-70 factor, ECF subfamily	3.81 (0.60)
**PGN 0319**	Probable RNA polymerase sigma-70 factor ECF subfamily	5.50 (0.87)
**PGN 0450**	Putative RNA polymerase sigma-70 factor ECF subfamily	2.88 (0.01)
**PGN 0970**	Putative RNA polymerase sigma-70 factor ECF subfamily	3.19 (0.23)
**PGN 0082**	Probable transcriptional regulator AraC family	-2.37 (0.33)
**9. GENES RELATED TO RIBONUCLEOSIDE TRIPHOSPHATE REDUCTASE**		
**PG 1260**	Anaerobic ribonucleoside triphosphate reductase	-2.54 (0.14)
**PGN 1396**	Anaerobic ribonucleoside triphosphate reductase	-2.28 (0.28)
**10. GENES RELATED TO RIBOFLAVIN**		
**PGN 0643**	3,4-dihydroxy-2-butanone 4-phosphate synthase	2.11(0.10)
**11. OTHER**		
***ung***	Uracil-DNA glycosylase	2.11 (0.06) ((0.06)
***vimF***	Virulence modulating gene F	-2.36 (0.25)
**PGN 1914**	Carboxyl-terminal processing protease	2.74 (0.23)
**PGN 1156**	Glycerol-3-phosphate dehydrogenase	-2.19 (0.06)
**PGN 0906**	Probable dihydoorate dehydrogenase electron transfer subunit	-2.26 (0.30)
**12. GENES RELATED TO HYPOTHETICAL PROTEIN**		
**PG 0100**	Hypothetical protein	2.78 (0.27)
**PG 0161**	Hypothetical protein	2.50 (0.49)
**PG 0215**	Hypothetical protein	4.72 (0.60)
**PG 0216**	Hypothetical protein	3.67 (1.14)
**PG 0217**	Hypothetical protein	3.01 (0.37)
**PG 0218**	Hypothetical protein	2.82 (0.41)
**PG 0323**	Conserved hypothetical protein	3.94 (0.45)
**PG 0606**	Hypothetical protein	2.43 (0.20)
**PG 0621**	Conserved hypothetical protein	-2.60 (0.24)
**PG 0622**	Hypothetical protein	-2.39 (0.12)
**PG 0986**	Hypothetical protein	2.99 (0.83)
**PG 1152**	Hypothetical protein	3.28 (0.95)
**PG 1267**	Hypothetical protein	2.46 (0.13)
**PG 1634**	Hypothetical protein	2.58 (0.68)
**PG 1675**	Hypothetical protein	2.55 (0.53)
**PG 1908**	Hypothetical protein	-2.08 (0.07)
**PG 2130**	Hypothetical protein	-2.50 (0.21)
**PG 2212**	Hypothetical protein	-9.49 (0.66)
**PG 2224**	Hypothetical proteinmembrane protein, putative	-2.84 (0.56)
**PGN 0052**	Hypothetical protein	2.33 (0.29)
**PGN 0078**	Hypothetical protein	-2.47 (0.25)
**PGN 0178**	Conserved hypothetical protein	-2.42 (0.31)
**PGN 0320**	Conserved hypothetical protein	4.11 (0.50)
**PGN 0321**	Conserved hypothetical protein	3.66 (0.28)
**PGN 0322**	Conserved hypothetical protein	3.31 (0.99)
**PGN 0323**	Conserved hypothetical protein	3.81 (0.20)
**PGN 0332**	Conserved hypothetical protein	2.33 (0.14)
**PGN 0486**	Conserved hypothetical protein	2.28 (0.12)
**PGN 0588**	Conserved hypothetical protein	-2.49 (0.35)
**PGN 0663**	Conserved hypothetical protein	-2.72 (0.26)
**PGN 0664**	Conserved hypothetical protein	-2.51 (0.58)
**PGN 0797**	Conserved hypothetical protein	2.15 (0.06)
**PGN 0837**	Conserved hypothetical protein	-2.30 (0.31)
**PGN 0907**	Conserved hypothetical protein	-2.91 (0.63)
**PGN 0969**	Conserved hypothetical protein	2.87 (0.23)
**PGN 1083**	Hypothetical protein	2.23 (0.09)
**PGN 1385**	Hypothetical protein	2.24 (0.05)
**PGN 1400**	Conserved hypothetical protein	2.65 (0.22)
**PGN 1639**	Conserved hypothetical protein	3.48 (0.46)
**PGN 1992**	Conserved hypothetical protein	-2.24 (0.22)
**PGN 2087**	Conserved hypothetical protein	-2.29 (0.12)
**uvrAll**	Conserved hypothetical protein	-6.89 (2.97)

^A^ Putative identification from Genebank.

^B^ Results of three biological replicates.

An additional group of genes related to oxidative stress and metabolism was differentially expressed in *P*. *gingivalis*, as shown in Table 2. This group of genes, represented by PGN_2076 and PG_2213, are involved in oxidative and/or regulatory mechanisms, as Nitric oxide (NO) stress resistance and were significantly suppressed in biofilm growth. These genes enable bacteria to survive within the inflammatory microenvironment of the periodontal pocket. Similarly, alkyl hydroperoxidase reductase subunits genes (AhpC-F (PG_0618, PGN_0660, PG_0619 and PGN_0661) were down regulated in *P*. *gingivalis* biofilms. These genes are involved in the primary defense against reactive oxygen species (ROS), and therefore affecting the bacterium aero-tolerance. In fact, PG_0619 was the gene most differentially suppressed (-13.13 (SD 0.70)). On the other hand, the putative genes related to metabolism NADPH-NAD transhydrogenases (PGN_1120, PGN_1122 and pntB) were up-regulated.

The genes involved in transposon functions, demonstrated heterogeneous results (Table 2). While genes corresponding to partial transposase in IS*Pg1* (PGN_0219, PGN_0575, PGN 1216 and PGN_1420) and PGN_0579 were down-regulated, genes belonging to the partial transposase in IS*Pg4* (PGN_0478) and PGN_0578 were up-regulated.

Genes related to the CRISPRs and associated CAS proteins system (CRISPR/Cas), like (PGN_1924-Cas2, PGN_1925-Cas1) were down-regulated in biofilm growth, while the gene PGN_1286, thought to be a lysozyme, was up regulated.

Among the genes related to fimbriae, only one gene, *fimD*, one of the minor components of the fimbriae A, appeared down-regulated by a factor of -2.30 (SD 0.26) in biofilm *versus* planktonic cells.

Among the genes involved in the biogenesis of components of ribosomal subunits, the genes *rpmH*, *rpsF* and *rpII* were up-regulated while *KsgA* were down-regulated when in comparing biofilm with planktonic growth.

The array data (Table 2) indicated that several RNA polymerase sigma factors of the σ70 family (PG_0214, PG_0985, PGN_0319, PGN_0450, PGN_0970), involved in the regulation of biofilm formation and diverse physiological processes, particularly virulence, were up-regulated in biofilm *versus* planktonic cells. On the other hand, PGN_0082, a probable transcriptional regulator in the AraC family, was down-regulated in biofilms cells.

The riboflavin-related gene encoded to the 3,4-dihydroxy-2-butanone 4-phosphate synthase/ GTP cyclohydrolase II protein (PGN_0643) was found up-regulated in *P*. *gingivalis* biofilm. This gene has been implicated in quorum sensing signaling and extracellular electron transfer. On the contrary, the gene VimF was down-regulated. This gene has been involved in the maturation/activation/anchorage of gingipains and other virulence factors of *P*. *gingivalis*. Lastly, 45% of the 92 differentially regulated *P*. *gingivalis* genes were of unknown or poorly characterized functions, most of them encoding unknown proteins.

**Fig 3:**
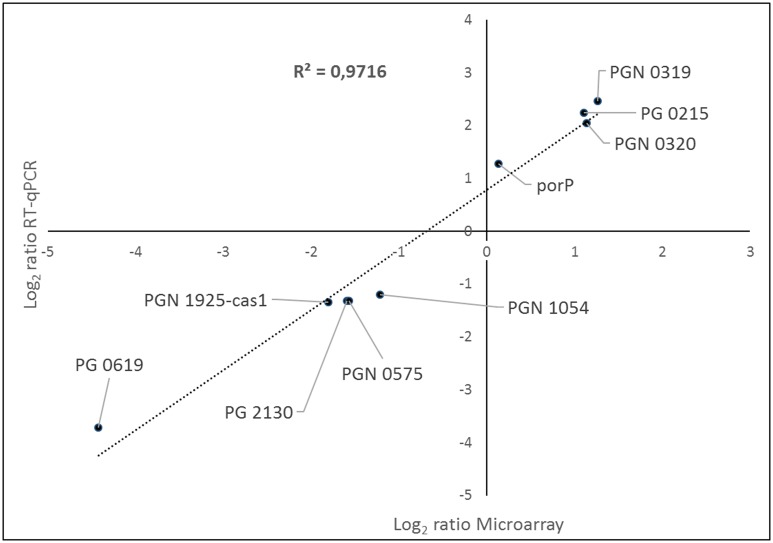
Correlation between microarray and Reverse Transcription-quantitative Polymerase Chain Reaction (RT-qPCR) gene expression ratios determined for biofilm *versus* planktonic cells. The RT-qPCR log_2_ values were plotted against the microarray data log_2_ values (R^2^ = 0.9716).

The microarray results were validated by RT-qPCR on four of the genes from the up-regulated group and five from the down-regulated group. Fig 3 illustrates the high correlation between the gene expression of logarithm-transformed of RT-qPCR plotted against the average log_2_ ratio values obtained by microarray analysis (R² = 0.9716).

## Discussion

This microarray-based comparative transcriptomic study has shown the up- and down- regulation of specific genes of *P*. *gingivalis* during the early stages of biofilm maturation (96 h of incubation). These gene expression patterns showed that 4.8% (92/1,909) of the genes of *P*. *gingivalis* significantly changed in biofilm, when compared to planktonic growth. Although this does not represent a huge difference between the two lifestyles [10, 20, 21], small changes in the level of expression of one gene can be amplified through regulatory networks and result in significant phenotypic alterations [22–24]. These results are in agreement with previous reports on other pathogens, such as *P*. *aeruginosa* or *Escherichia coli* grown under similar differential growth conditions, in which less of 5% of differential expression was demonstrated [10, 11, 25, 26].

When assessing the different functional categories affected by the differentially regulated genes, a wide diversity was observed, which may indicate that the adaptation of *P*. *gingivalis* to a community lifestyle required a broad-based transcriptional modulation. This adaptation involved different virulence factors, as proteins codifying for outer membrane proteins or for fimbriae. Outer membrane vesicles (OMVs) of *P*. *gingivalis*, which are formed by “blebbing” portions of their outer membrane, have been recognized as important virulence factors of this pathogen in relation to periodontitis [6]. These vesicles contained specific proteases, termed gingipains (Arg-gingipain [Rgp] and Lys-gingipain [Kgp]) [5] associated with the capacity of *P*. *gingivalis* to invade host epithelial cells [27, 28]. This transcriptomic study has revealed four genes, which codify proteins located in the OMVs of *P*. *gingivalis* being over-expressed (OmpH-1 and OmpH-2, PG_0448, and PG_0987). This finding was already described by Veith *et al*. (2014) [29]. Similarly, Kuboniwa *et al*. (2009) [30] using proteomic technology studied *P*. *gingivalis* in biofilm growth and reported significantly increased cell envelope proteins, such as OmpH protein PGN_0301, whose encoding gene has been shown over expressed in this investigation.

The up-regulation of these proteins in biofilm *versus* planktonic state has also been reported in others studies demonstrating that OMVs and related genes play an important role in bacterial co-aggregation [31] and attachment to epithelial cells [32]. Although differential expression of genes has been shown at *in vivo* polymicrobial biofilms (Díaz and Kolenbrander [33], this study has confirmed that up-regulation could also occur when growing in an *in vitro* mono-species *P*. *gingivalis* biofilm.

Fimbriae of *P*. *gingivalis* have also been recognized as a major virulence factor, since they mediate in cell adhesion and may facilitate their capacity to invade periodontal tissues [34–38]. Only one gene, *fim D*, was found down-regulated in this study. This gene is a minor component of a seven gene cluster, *fimX*, *pgmA and fim ABCDE*, which encode type 1 fimbriae, and it is characterized by mannose-sensitive hemagglutination and being assembled via the chaperone/usher pathway [39, 40]. These genes participate in the biogenesis of the fimbriae, regulating their number and length, as well as their adherence function [41, 42]. Nevertheless, Krogfelt and Klemm (1988) observed that a clone of *E*. *coli*, not containing the genes encoding the minor component proteins, still produced fimbriae consisting of pure Fim A protein, (main structural component of the fimbriae type I), indicating that, at least in the case of *E*. *coli*, the minor components were not necessary for the structural integrity of the fimbriae, although these fimbriae were non-adhesive and did not confer hemaglutination [42–45]. Similarly, Whiteley *et al*. (2001) suggested that these appendages may not be required at the later stages of biofilm formation for maintenance of a mature biofilm, since fimbria, pili or flagella were only involved at initial steps of attachment [10, 15].

The lipoprotein-related gene PGN_0151 was over-expressed in biofilm. Hirano *et al*. (2013) reported that a mutant of this gene was reduced in its ability to form biofilms compared to wild type [46] what suggests that these genes were significantly involved in the biofilm lifestyles of *P*.*gingivalis*. In regards to those genes involved in the adaptation to new local environmental conditions, this investigation showed a differential expression of those genes involved in the transposition system (PGN_0219, PGN_0575, PGN 1216, PGN_1420, PGN_0579, PGN_0478 and PGN_0578), some of them codifying insertion sequences (IS). Since transposition is generally known to be triggered by cellular stress [47–49], this finding suggests that these transposable elements, moving from one site within the genome to another, could have an important role in the genomic re-arrangement and recombination in *P*. *gingivalis* growing in biofilm. This adaptation to stressful local environmental conditions has been previously reported [7, 50–53]. Furthermore, the CRISPR-Cas and associated CAS proteins system represents a unique system that provides prokaryotic cells, as *P*. *gingivalis*, adaption and protection from host defenses [54, 55]. Down-regulation of the genes PGN_1924-Cas2, PGN_1925-Cas1 may suggest a decrease in the defensive capability of *P*. *gingivalis* ATCC 33277 when growing as single-species biofilm *in vitro* or its adaptation to an environment without competing species.

Gene PG_2213, encoding a putative nitrite reductase-related protein and implicated in nitric oxide (NO) stress resistance was repressed in *P*. *gingivalis* biofilm growth [56, 57]. The ability to down-regulate nitrite reduction [58], involves the expression of several genes known to be induced by nitrogen oxides and low oxygen tension [59, 60]. Whether *P*. *gingivalis* PG_2213 has a similar role is unknown. Boutrin et al. (2012) suggested that NO stress resistance in *P*.*gingivalis* was facilitated by a complex and tightly regulated network of genes involved in multiple pathways, including, energy metabolism, gene regulation, detoxification, and virulence [56].

Furthermore, although *P*. *gingivalis* seems to lack a protective NADH oxidase, Alkyl hydroperoxide reductase (genes PG_0618, PGN_0660, PG_0619 and PGN_0661), C subunit (AhpC), have been reported to be involved in *P*. *gingivalis* aero-tolerance processes. The up-regulation of genes related to NADPH-NAD transhydrogenases (PGN_1120, PGN_1122, *pntB*) suggests that *P*. *gingivalis* growing in biofilm has elevated metabolic activities, as shown with *C*. *acetobutylicum*, by Liu *et al*. (2016) [9]. In this investigation, several genes related to ribosome function (*rpmH*, *rpsF*, *rpII* and *KsgA)* were over expressed in the biofilm, what may indicate that the metabolic increase was associated to ribosome function, that may require up to 40% of the cell's energy in growing bacteria [52].

The observed differential up regulated expression of sigma factors in biofilm cells (PG_0214, PG_0985, PGN_0450, PGN_0970, PGN_0319) might indicate that these genes are important regulators of *P*. *gingivalis* during biofilm growth [8]. Similar results have been reported for *E*.*coli* [61]. Besides, members of the AraC family of transcriptional regulators (PGN_0082), with decreased expression in the biofilm, have been shown to be important in carbon metabolism (degradation of sugars such as arabinose), stress response to virulence in other species [62], and in the regulation of quorum sensing signaling in *P*. *aeruginosa* [63]. Also, related to quorum sensing signaling, the up regulated gene PGN_0643, has been involved in the biosynthesis of riboflavin, a substance associated in a number of extracellular processes by bacteria, especially Gram-negative organisms [64–66].

There are, however, important limitations associated to this study, since the biofilm used was an *in vitro* single-species model. The obtained results, however, may serve as a resource for future studies in oral biofilms aimed to further understand the genetic basis of the regulatory mechanisms of *P*.*gingivalis* and other pathogenic bacteria involved in subgingival biofilm growth and maturation.

## Conclusions

By means of transcriptomic analysis, this study has shown that 4.8% of the *P*. *gingivalis* ATCC 33277 genome exhibited differential expression profiles when grown in biofilm. In such biofilm growth, the up-regulated genes were mainly those related to the cell envelope, as the genes encoding for the cationic OMPs or gene PGN_0151, which appear as a novel *P*. *gingivalis* gene that seems to have a role in the biofilm state. Also, the genes implicated in PorSS system and RNA polymerase sigma factors of the σ70 family, which are genes related to virulence/proliferation factors were up-regulated. On the contrary, the expression of most of the genes involved in oxidative stress or CRISPRs system were suppressed.

Therefore the adaptive response of *P*. *gingivalis* in biofilm growth demonstrated changes in gene expression profiles.
